# Body condition of stranded Razorbills and Atlantic Puffins in the Western Mediterranean

**DOI:** 10.1002/ece3.70161

**Published:** 2024-09-11

**Authors:** Yada Trapletti‐Lanti, Mónica Expósito‐Granados, Sergio López‐Martínez, Miguel Torres, Marga L. Rivas

**Affiliations:** ^1^ Department of Biology, Institute of Marine Science INMAR University of Cadiz Cadiz Spain; ^2^ Department of Biology and Geology University of Almería Almería Spain; ^3^ Department of Animal Sciences, Faculty of Sciences Universitat Politècnica de València Valencia Spain

**Keywords:** body condition assessment, conservation, mass die‐off events, metabolic analysis, stranded seabird

## Abstract

Annual mass migrations of seabirds between their breeding and wintering grounds are critical for ensuring their survival and reproductive success. It is essential to comprehend their physical condition in order to identify the causes of death and to facilitate conservation efforts. This study focuses on evaluating the age, body condition index, and metabolites in liver and muscle (triglycerides, glycerol, glycogen, cholesterol, lactate, and glucose) of stranded Razorbills (*n* = 84) and Atlantic puffins (*n* = 11). The study was conducted along the Andalusian coast of Spain during the winter season of 2022–2023. The study examined the body condition of stranded individuals and their metabolic state to determine potential factors that may have caused their deaths. The study found that the majority of stranded individuals were juveniles. Both species exhibited low levels of carbohydrate (glucose and glycogen) in their tissues and high levels of lactate in their muscles. These findings could suggest that the individuals had undergone prolonged, strenuous exercise, demanding energy on anaerobic pathways, which may have been associated with migration. The study highlights the significance of adhering to standardized protocols when assessing the body condition of stranded seabirds. Doing so can help to identify causes of death and facilitate conservation efforts. A proposed index for body condition, which incorporates biometric measurements and individual physical condition, provides a comprehensive means of understanding the health of these unique species. This study underscores the importance of further research into the conservation measures and recommendations for protecting seabird populations. It is critical to comprehend the contributing factors of mass mortality incidents to work towards safeguarding these species and preserving their vital migration patterns.

## Graphical abstract

The study evaluates mass mortality in the family Alcidae in the western Mediterranean Sea by analysing the body condition and metabolic status of stranded seabirds. A new index is proposed to globally estimate the condition of dead specimens, finding that most are juvenile birds with low carbohydrate and high tissue lactate levels.

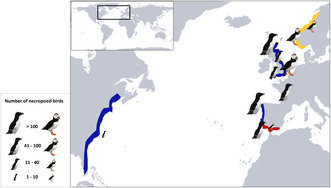

## 1 INTRODUCTION

The annual migration between breeding and wintering grounds is a common behaviour that is found in a range of taxa, including many bird species (Winger et al., 2019). Differences in migration strategies, wintering destination, and timing can significantly impact survival and subsequent breeding success (Fayet et al., 2016). Several factors play a role in migration, including condition on their breeding grounds previous migration, which affects the condition of adults and juveniles previous starting the migrations. The physical condition of both adult and juvenile individuals is also vital for the success of their migrations (Steenweg et al., 2022). As well as climatic phenomena and exposure to threats during migration, or the experience of the individuals (Oro, 2014). For instance, the North Atlantic Oscillation (NAO) plays a crucial role in influencing the migration patterns of birds between Europe and the Mediterranean region (Hüppop & Hüppop, 2003). Long‐term studies conducted at breeding sites have shown a strong relationship between the availability and quality of food resources and the reproductive output of seabirds (Grémillet et al., 2004). Additionally, fat reserves are likely to influence the success of their migrations (Goymann et al., 2010). Changes in juvenile migration routes have also been identified as a contributing factor to mortality during migration episodes (Lane et al., 2021). In winter 2022–2023 there was a mass stranding event, in which the majority of species stranded in the coast of the western Mediterranean included Razorbill (*Alca torda*) and Atlantic puffin (*Fratercula arctica)*, which belong to the Alcidae family, also known as Auks. These birds are adapted for a marine lifestyle, with streamlined bodies, webbed feet, and wings designed for swimming and diving (Schreiber & Burger, 2001). These animals usually nest in specific regions, such as islands, coastal areas with cliffs, or loctions that provide protection from predators due to their geomorphology (Lloyd, 1979). They feed a wide variety of prey, including small pelagic fish, mesopelagic fish, demersal fish, invertebrates, plankton, and carrion (Provencher et al., 2019). They are primarily considered North Atlantic species, with breeding and wintering populations concentrated in regions such as the coasts of North America, northern Europe, and the Arctic. In Europe, these North Atlantic seabirds winter in the North Sea (Stone et al., 1995; Del Nevo et al., 2013) and breed in several locations, such as East and West Britain, Ireland, North Scotland, Norway, and Iceland, hosting important colonies (Harris, 1984; Harris et al., 2010; Mitchell et al., 2004).

While Razorbill and Atlantic puffin are not typically considered regular migrants to the Mediterranean Sea or the coasts of Portugal, studies have identified wide dispersive migration and strong fidelity routes (Fayet et al., 2016). Furthermore, high mortalities have been recorded at southern latitudes, such as Portugal (Teixeira, 1986) and Spain (Morley et al., 2016). These mortalities could be attributed to various factors, including extreme weather conditions such as exposure to storms and winter cyclones, leading to starvation (Clairbaux et al., 2021). Some authors, such as Marchetti and Price (1989) and Wunderle (1991), have demonstrated that juvenile bird species sometimes have a lower probability of survival due to factors such as lower foraging proficiency, inexperience, or physical skills. This vulnerability, coupled with the decline in forage fish species, may contribute to population decreases among the species studied during their juvenile stages (Kress et al., 2016). Therefore, it has been predicted that stranded individuals are mostly juveniles, as juveniles are more likely to die during the migration, as shown in other alcid species (Lane et al., 2021; Robinson et al., 2020). However, it is also important to consider that seabirds wintering and feeding in the North Atlantic Ocean may play a role in determining whether an energetic steady state is reached (Fort et al., 2009). Winter foraging conditions may influence subsequent breeding performance and, consequently, body condition, and fitness of migrating seabirds (Furness et al., 2006).

Necropsies of stranded seabirds can assess the condition of their muscles, fat deposits, and overall body condition, providing insights into their state before death. The body condition index is usually measured to gain insights into the health, nutritional status, and overall well‐being of birds. This index provides critical information about a bird's physiological state, which can be particularly useful in understanding the condition of birds before death, indicating their energy reserves, or helping to determine if starvation, disease, or other factors contributed to mortality (Labocha & Hayes, 2012). In this sense, knowledge of energetic metabolism through tissue analyses of biochemical parameters related to carbohydrate and lipid metabolism can be useful in understanding the metabolic conditions of collected birds and forecasting mortality causes. Under stressful circumstances such as fasting or inclement weather, birds activate the stress system (Bohler et al., 2021; Siegel, 1980), showing different physiological reactions that increase metabolic energy demands to maintain activity and recover cellular homeostasis (Griminger, 1986; Hazelwood, 1986). Among biomarkers, the presence of lactate and mobilization of glucose and glycogen are commonly associated with intense exercise and fatigue, especially in muscle and liver, where these compounds are synthesized and accumulated (Bird et al., 2014; Gladden, 2004). On the other hand, lipids are essential macromolecules for animals, playing vital roles in biological processes such as energy supply, integrity of biological membranes (phospholipid bilayers), and signalling molecules (hormones) (Sargent et al., 2003). Similarly, triglycerides and cholesterol are important lipid components for energy storage, cellular integrity, and tissue functionality in animals, particularly in birds (Blem, 1976), due to their energetic role and the presence of fatty acids (Ω3 and Ω6) in their composition (Fraser, 1974; Sargent et al., 2003). In summary, evaluating the values of biochemical parameters such as cholesterol, triglycerides, lactate, glucose, and glycogen in muscle and liver can provide valuable information about its energy status, metabolic condition, physical performance, nutritional status, and possible pathologies. However, information on the body condition and metabolic measurements in stranded Razorbills and Atlantic puffins is very limited (Anker‐Nilssen et al., 2018; Diamond et al., 2020; Simpson & Fisher, 2017). Only two studies specifically reported fat deposition (Anker‐Nilssen et al., 2003) or scoring of muscle, subcutaneous, and intestinal fat (Anker‐Nilssen et al., 2017). Therefore, there is a need for standardized protocols for assessing the body condition of Razorbills and Atlantic puffins.

In this line, the aims of the study are: (i) to characterize mortality of the Razorbill and the Atlantic puffin in terms of species and age in the western Mediterranean Sea; (ii) to describe the body condition of these seabirds and determine their state at death by using body mass index and metabolic measurements and (iii) to compare the obtained results of Razorbill and Atlantic puffin species with the global mortality rates, specific necropsy procedures, and the assessments of their physical state and potential causes of death.

## 2 METHODOLOGY

### 2.1 Study area and species

**FIGURE 1:**
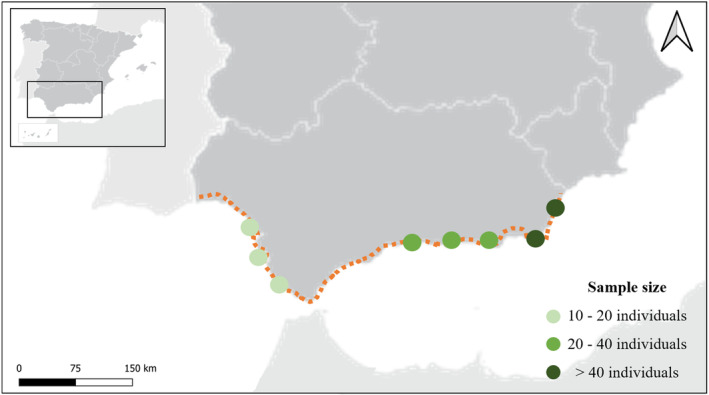
Study area of the seabirds' collection (marked with the dashed orange line). Dots indicate sample sizes in each area. Data were collected from November 2022 to March 2023.

The study area was the southern coastline of Spain, located to the west of the Mediterranean Sea (Figure 1). The species studied were the Razorbill and the Atlantic puffin, both belonging to the Alcidae family. According to the IUCN (2022), they are classified as ‘least concern’ and ‘vulnerable’, respectively.

### 2.2 Ethical statement and sample collection

In the winter season, from November 2022 to March 2023, a total of 703 stranded alcids were reported. A subsample of 95 carcasses was selected for further analyses, of which 84 were Razorbills and 11 were Atlantic puffins. Of these, only 86 individuals (76 Razorbills and 10 Atlantic puffins) could be analysed, as individuals that were not fresh were removed in order to avoid any alteration to the results. The main sources of carcasses were (i) stranded seabirds on beaches reported by volunteers and collected by researchers (*n* = 87) and (ii) seabirds stored in wildlife recovery centres (*n* = 8) collected in the same season. The individuals were stored in freezers at −20°C until the necropsies could be carried out. The collection of carcasses and tissue analysis has been carried out following authorization and ethical approval from the Ministry for Ecological Transition and the Demographic Challenge (MITECO, Spanish Government, Ref: SGBTM/BDM/AUTSPP/17/2022), and from Junta de Andalucía (Councillor for Sustainability, Environment, and Blue Economy).

### 2.3 Sample processing

#### 2.3.1 Body condition index

To estimate the possible cause of death, necropsies were conducted following the dissection protocol outlined by van Franeker (2004). Data of biometric variables including body mass, wing length, tarsus length, head length, culmen length, bill length, bill width, and age category (adult or juvenile) of each individual were collected (Table S1).

To calculate the age of the individuals, we applied three criteria as reported by Espín et al. (2010), such as bill development (Anker‐Nilssen, 1988), the size, and appearance of the gonads (Camphuysen & Van Franeker, 2007; van Franeker, 2004), and the presence and dimensions of the bursa of Fabricius (Camphuysen & Van Franeker, 2007).

To calculate the body condition index, the skin was carefully peeled off on each side while keeping the fat layer attached to the skin. The condition of the pectoral muscles was visually assessed and scored as follows: 0 (strongly emaciated), 1 (emaciated), 2 (moderate condition), and 3 (good condition). The quantity of subcutaneous and intestinal fat was classified as 0 (no fat), 1 (some fat), 2 (fat), and 3 (very fat) (Figure S1). The body condition (BC) index was calculated as the ratio of wing length with body mass along to the sum of the subcutaneous fat score (SF), the intestinal fat score (IF), and the pectoral muscle score (M), as shown in Equation 1.

(1) $$\mathrm{BC}\;\text{index}=\left(\frac{\text{Wing length}}{\text{Body mass}}\right)+\mathrm{SF}+\text{IF}+M$$

Equation 1. Proposed Body Condition Index.

#### 2.3.2 Metabolic condition

Between 0.1 and 0.2 g of muscle and liver were collected from Razorbill and Atlantic puffin. The muscle and liver samples were then snap‐frozen in liquid nitrogen and stored at −80°C until further biochemical analysis.

Frozen samples used for the analyses of metabolites were mechanically homogenized, following the procedure described in Molina‐Roque et al. (2022). Metabolites in tissues were quantified using commercial kits (SpinReact SA, St. Esteve d' en Bas), with reactions adapted for 96‐well microplates. The metabolic parameters were spectrophotometrically analysed in duplicate in each tissue sample of both bird species using a PowerWave™ 340 microplate spectrophotometer (BioTek Instruments). The levels of glucose (Glucose‐HK Ref. 1,001,200) and glycogen were estimated using the method described by Keppler (1974), while amyloglucosidase (Sigma‐Aldrich, Ref. A7420), lactate (Lactate Ref. 1,001,330), cholesterol (Cholesterol Ref. 1,001,091) and triglycerides (TAG Ref. 1,001,311) were quantified according to the procedure outlined by Perera et al. (2020).

### 2.4 Literature review of global dataset

We conducted a literature review in the field of stranded Razorbills and Atlantic puffins. The dataset was obtained by searching for scientific articles indexed in the Scopus database up to September 2023. The search string included different terms related to the taxonomic group (‘alcid*’, ‘alca torda’, ‘fratecula artica’, ‘Atlantic Puffin*’, ‘Razorbill*’) and terms related to body condition or physical state (‘body condition’, ‘mortality’, ‘necropsy’). The search was applied to titles, abstracts, and keywords, initially returning a total of 1200 published articles. We then restricted the search based on selection criteria, choosing articles that reported mortality, necropsies, or mass stranded events in one or both of the two species under study. Subsequently, a total of 76 full articles were evaluated, of which 9 were finally selected for inclusion in this study. Articles that did not conduct post‐mortem examinations, those that reported instances of mortality caused by oil spread or fishing gear, and those that conducted post‐mortem examinations but only evaluated the presence of pollutants were excluded. These 9 articles, published worldwide, were combined with our own data to create a comprehensive database spanning from 1989 to 2023. This dataset included a total of 1695 stranded seabirds of Razorbill (*n* = 1401) and Atlantic puffin (*n* = 294) species (Table S2).

In each publication, we extracted data on stranded seabird necropsies in each species, geographic location, study date, number of individuals analysed, sample processing, and identification and analysis technique of body condition (Table S3).

### 2.5 Statistical analysis

Statistical analysis was conducted using the R Studio package, version 4.4.0 (RStudio Team, 2023). Non‐parametric methods were employed for statistical analysis after the data was determined to be abnormally distributed through a Shapiro‐Wilk test. Moreover, the Kruskal‐Wallis test was performed with a significance level of 0.05 to compare the biometric variables between species. In this test, we included the variables of body mass, wing length, tarsus length, head length, culmen length, and bill length.

Biochemical parameter values from muscle and liver were checked for normality and homogeneity of variance using Kolmogorov‐Smirnov and Levene's tests, respectively. Data were analysed by an independent sample *T*‐student test (*p* ≤ .05). The results are presented as means ± SD.

To propose a standardized global body condition index for stranded seabirds, several analyses were conducted. Initially, a principal component analysis (PCA) was performed to identify patterns and relationships among various variables collected from necropsies. To enhance the robustness of our dataset, we also conducted a Random Forest analysis to assess the correlation of each variable with ‘body mass’, which is a critical component of the body condition index.

## 3 RESULTS

### 3.1 Stranded alcid seabirds in the Western Mediterranean

Razorbills (*n* = 84) were mostly juveniles (84.5%); the mean weight was 411.89 ± 52.31 g, the wing length measured 188.90 ± 4.58 mm, and the tarsus length was 31.36 ± 1.69 mm. No significant differences were found in the measurements except for wing length. Adults had an average 5.7 mm larger wing length than juveniles. Among the Atlantic puffins, most of the individuals were juveniles (63.6%). In the complete sample (*n* = 11), the average weight was 257.43 ± 91.51 g, the wing length measured 168.95 ± 30.83 mm, and the tarsus length was 27.30 ± 2.20 mm. Other variables such as head length, culmen length, and bill width and length were also measured (Tables S3 and S4).

### 3.2 Body condition

**FIGURE 2:**
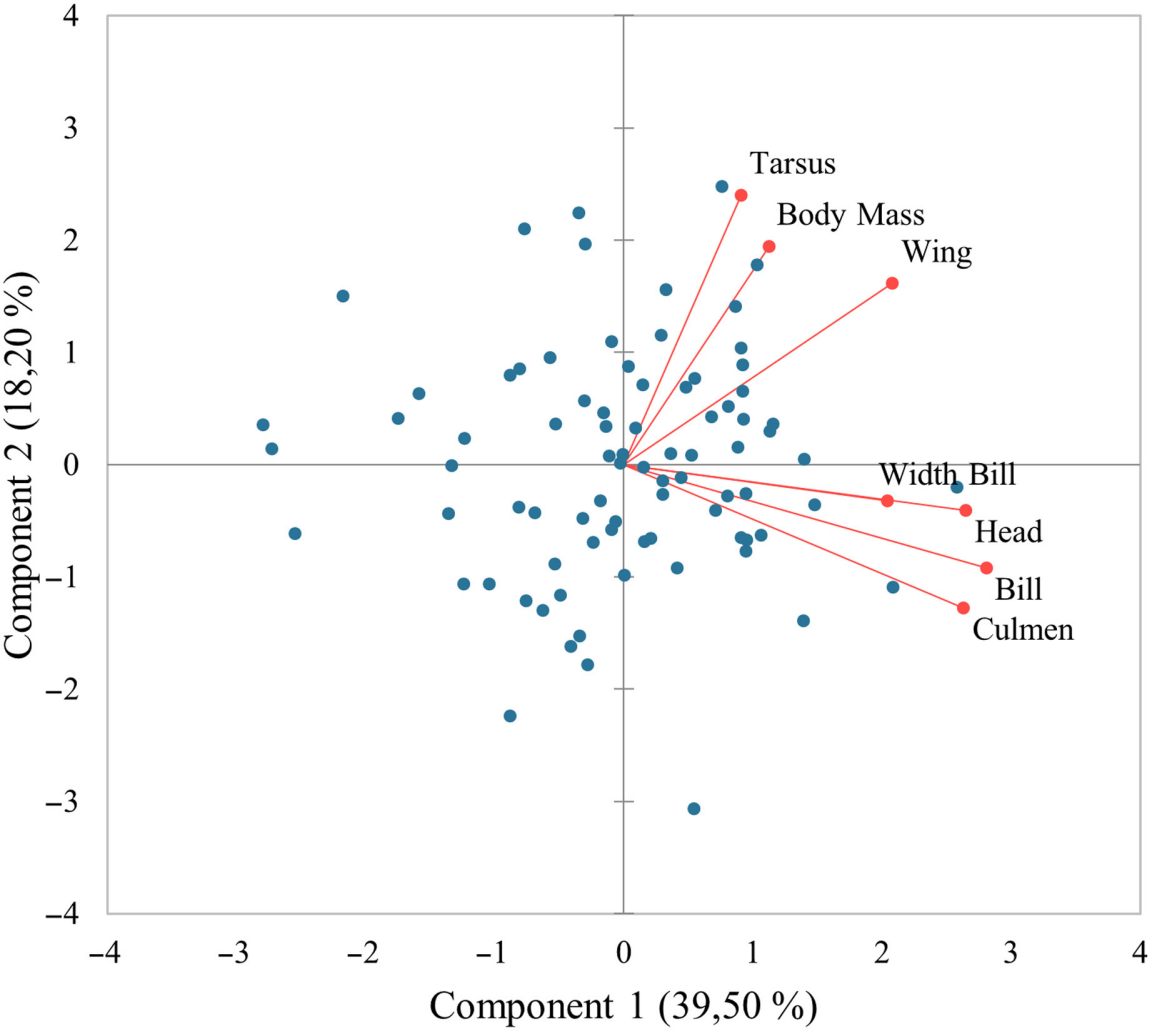
PCA for Razorbill: Variable Distribution and Relationships. The axes represent the principal components, with captures the most variance in the data. Red lines represent the relationship of each variable to the principal components. Quadrants indicate the relationship between observations and principal components. (0–4) positive influence on directions, (0–4) negative influence on directions.

During the PCA, we identified the variables that constituted the initial two factors, which accounted for over half of the sample (57.69%) (Figure 2). Specifically, Component 1 explained 39.50% of the sample variation and consisted of the measurements of bill, head, and culmen, while Component 2 explained 18.20% of the sample variation and encompassed the measurements of tarsus, body mass, and wing (Table S5 and Table S6).

As shown in Table S7, the variable most highly correlated with weight in the random forest analysis, excluding the variables related to bill measurements, was wing length. Bill measurements were not selected for further analysis because they cannot be standardized across species, as they are also correlated with individual growth. Therefore, we chose two variables from the second factor with the most significance, such as body mass and wing length.

**FIGURE 3:**
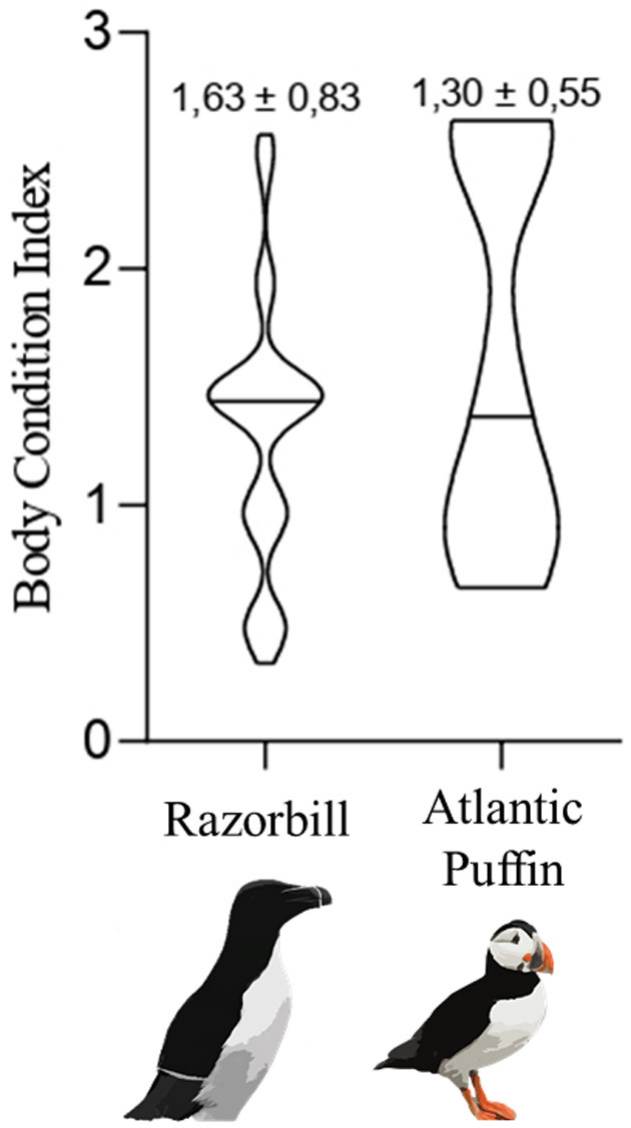
Violin plots of Body Condition Index (calculated using wing length, body mass, subcutaneous fat, internal fat, and pectoral muscle) in Razorbill (*n* = 76) and Atlantic puffin (*n* = 10). The black line is the mean of body condition, and the width of the violin represents the sample size.

When comparing the body condition index of each species, no significant differences were observed between Razorbills (*X* = 3.151, *df* = 1, *p* = .075) and Atlantic puffins (*X* = 0.617, *df* = 1, *p* = .432) (Figure 3).

### 3.3 Metabolic condition

**FIGURE 4:**
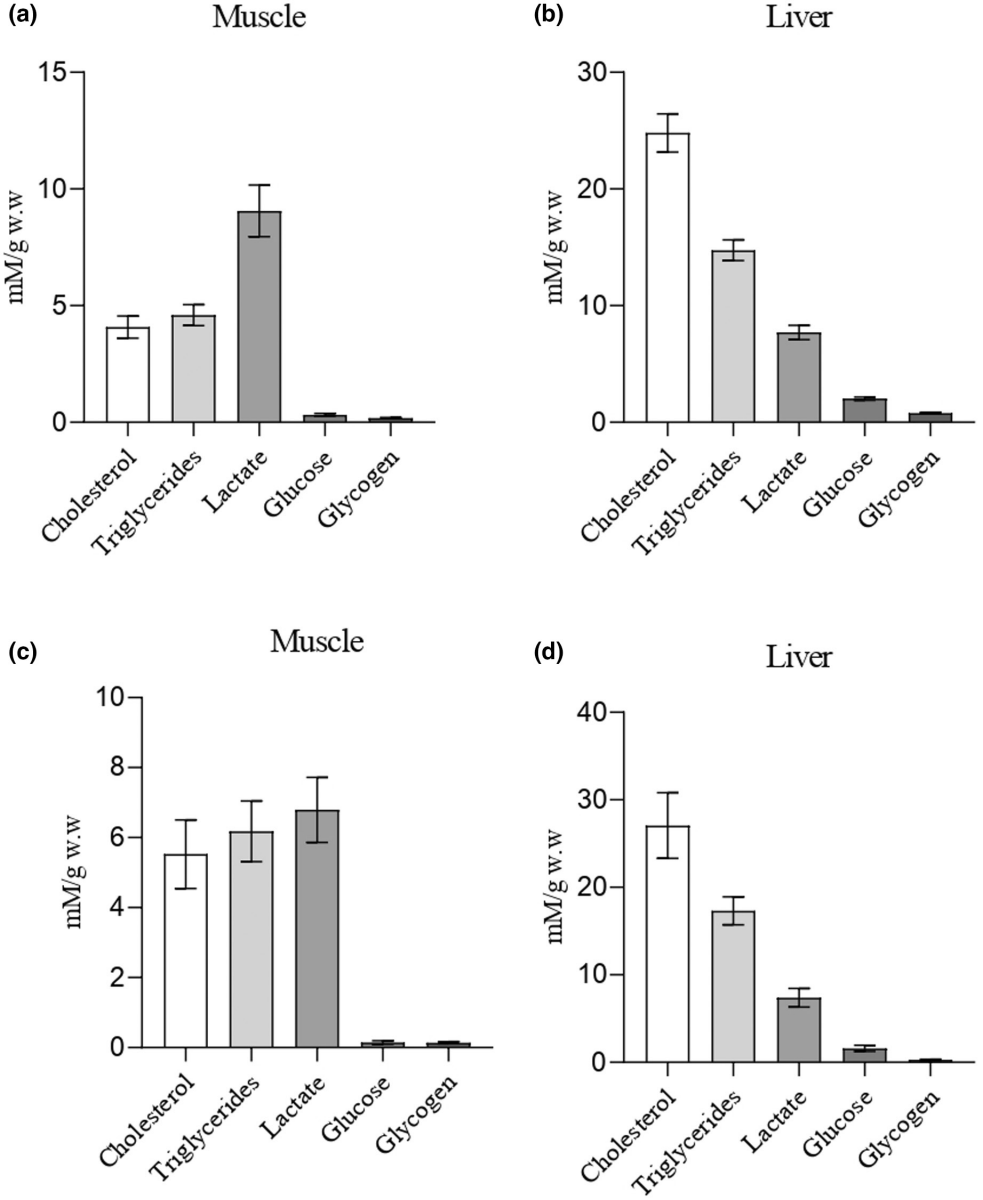
Bar plots of tissue metabolic condition of muscle and liver of Razorbill (a,b; *n* = 76) and Atlantic puffin (c,d; *n* = 10). mM/g w.w (millimoles/grams of wet weight). The bar represents the standard deviation.

Both species exhibited minimal levels of glucose and glycogen in the analysed muscle and liver tissues (Figure 4). Moreover, in both species, muscle tissue showed elevated levels of lactate (Figure 4a,c), while the liver exhibited significant levels of cholesterol and triglycerides, with cholesterol being the main predominant lipid (Figure 4b,d).

**FIGURE 5:**
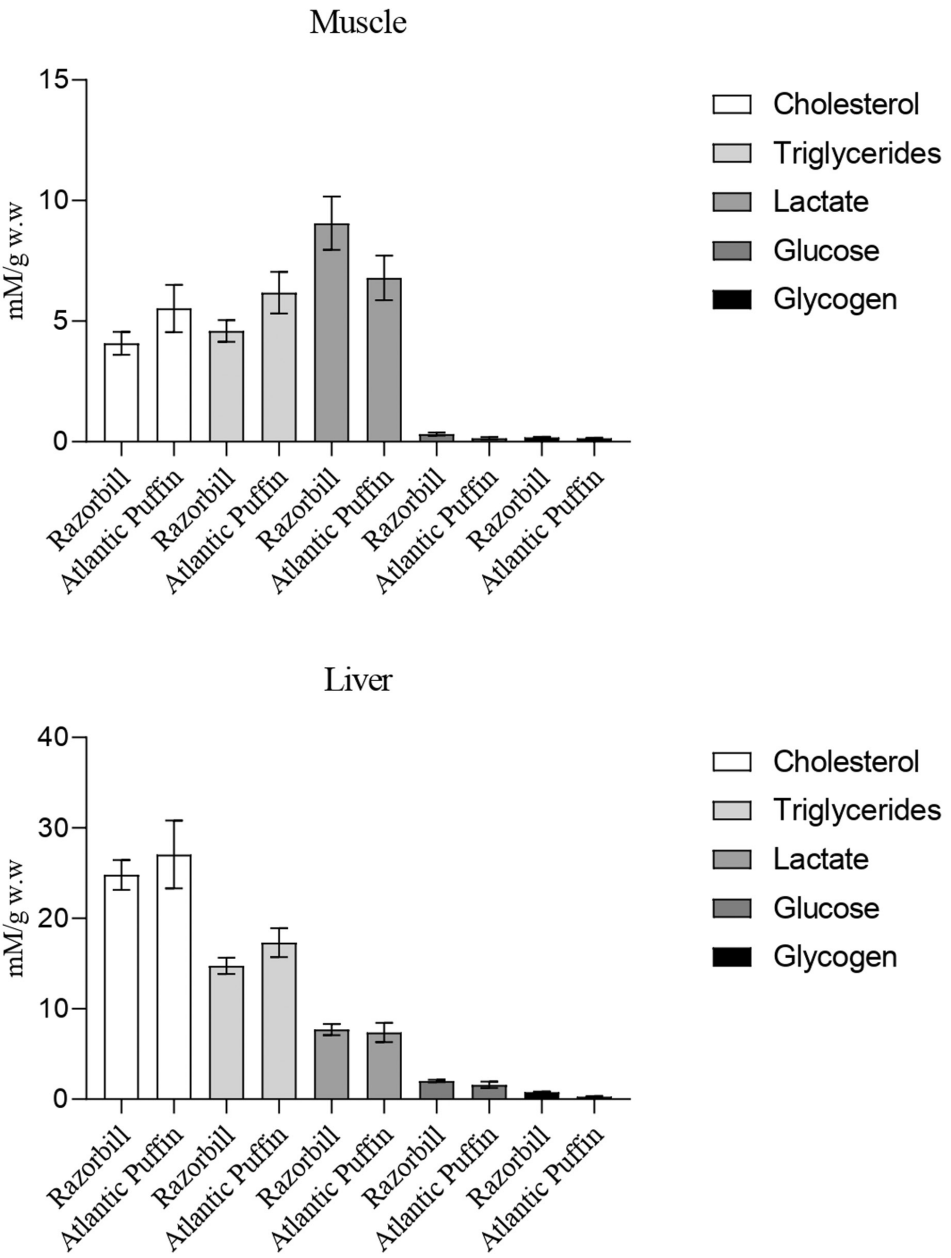
Bar plots of tissue metabolic condition of muscle and liver of Razorbill (*n* = 76) and Atlantic puffin (*n* = 10). mM/g w.w (millimoles/grams of wet weight).The bar represents the standard deviation.

**FIGURE 6:**
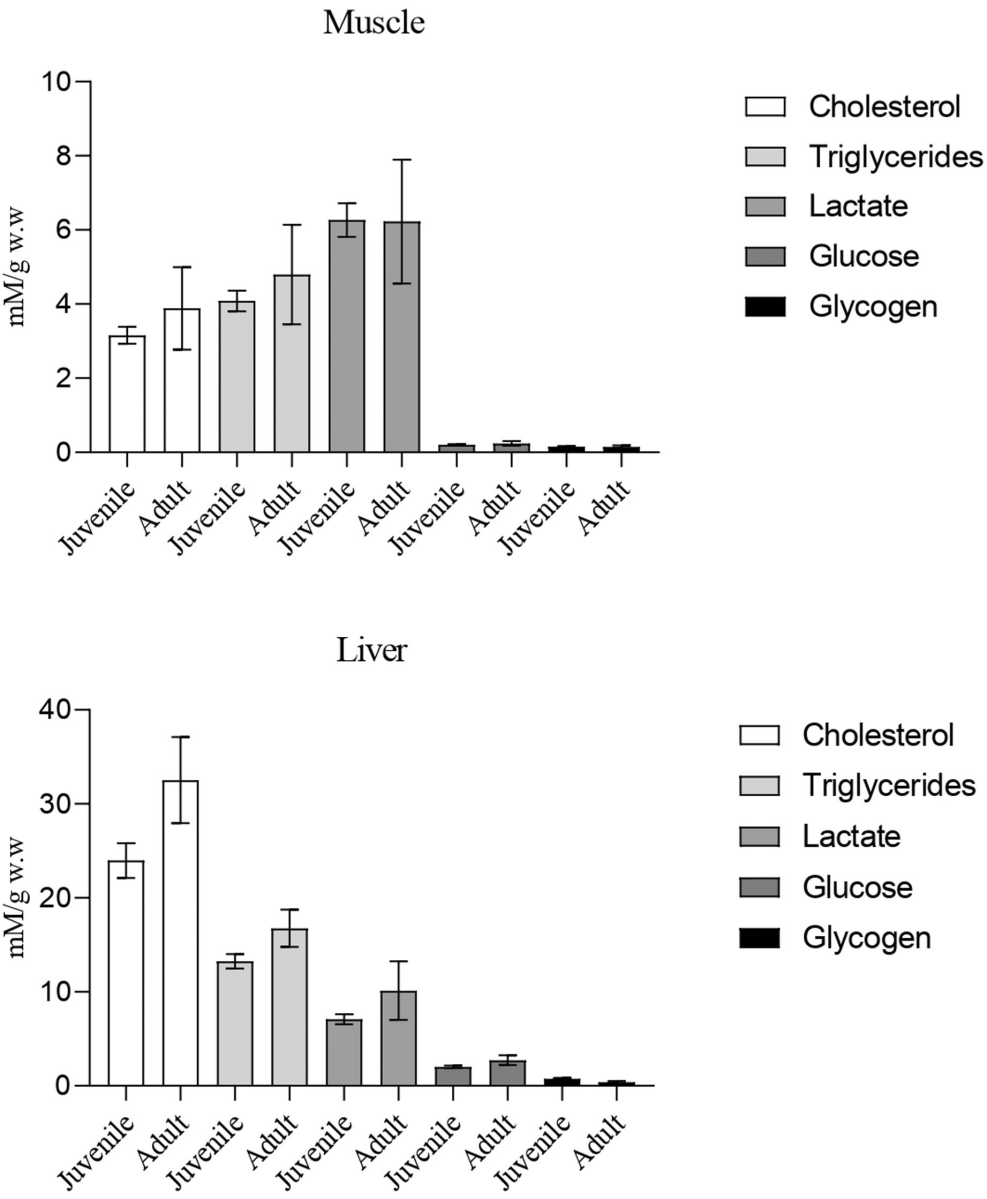
Bar plots of tissue metabolic condition of muscle and liver between juvenile (*n* = 57) and adult (*n* = 8) individuals of Razorbill. mM/g w.w (millimoles/grams of wet weight).The bar represents the standard deviation.

**FIGURE 7:**
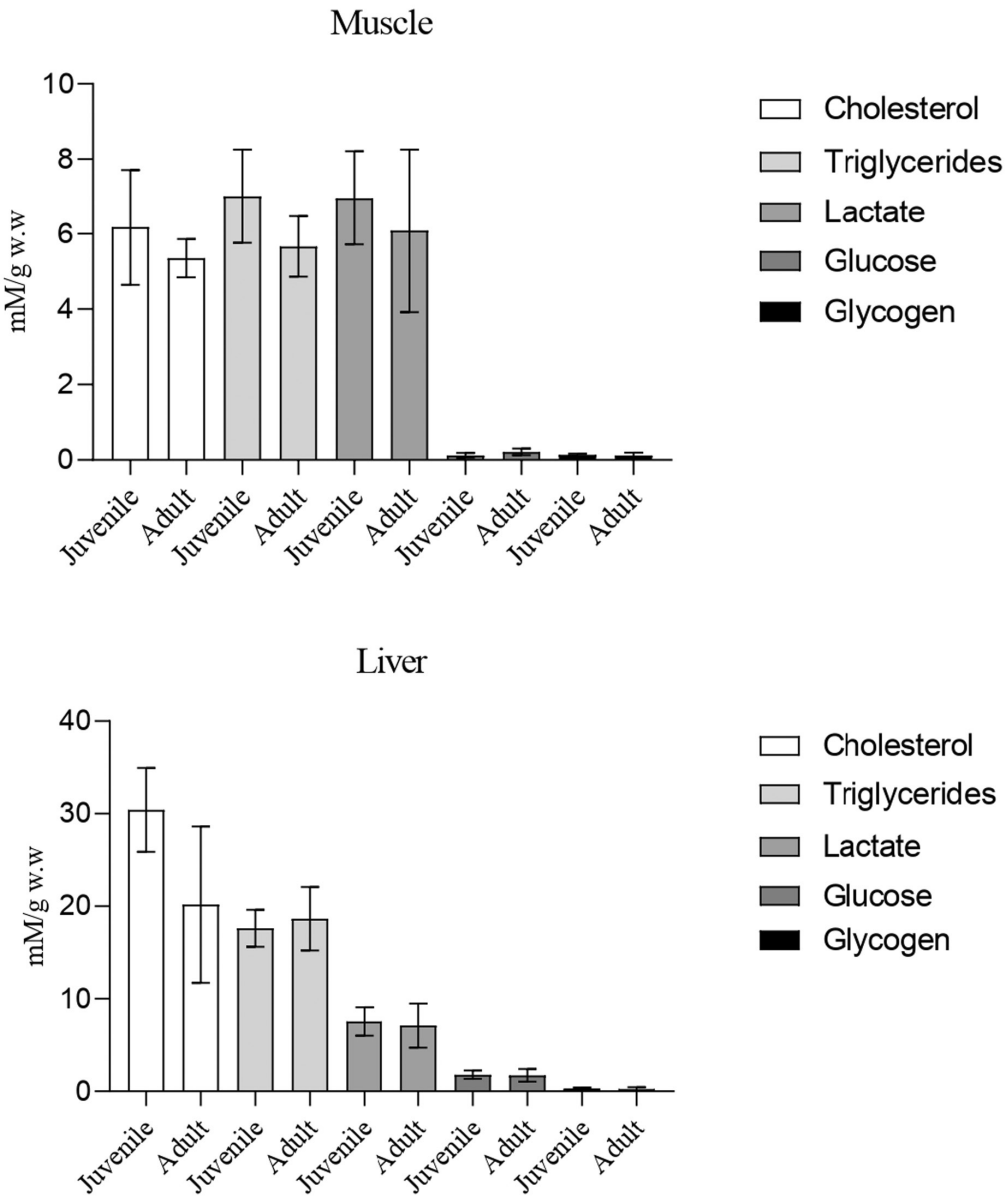
Bar plots of tissue metabolic condition of muscle and liver between juvenile (*n* = 6) and adult (*n* = 3) individuals of Atlantic puffin. mM/g w.w (millimoles/grams of wet weight).The bar represents the standard deviation.

Analyses between metabolic condition on muscle and liver of Razorbill and Atlantic puffin denoted the absence of significant differences (*T*‐test; *p* < .05) between species for the compounds analysed (Figure 5). Similar results are found when metabolic data from tissues are compared, differentiating between juveniles and adults of Razorbill (Figure 6) and Atlantic puffin (Figure 7), since no significant differences were detected (*T*‐test; *p* < .05).

### 3.4 Literature review

**FIGURE 8:**
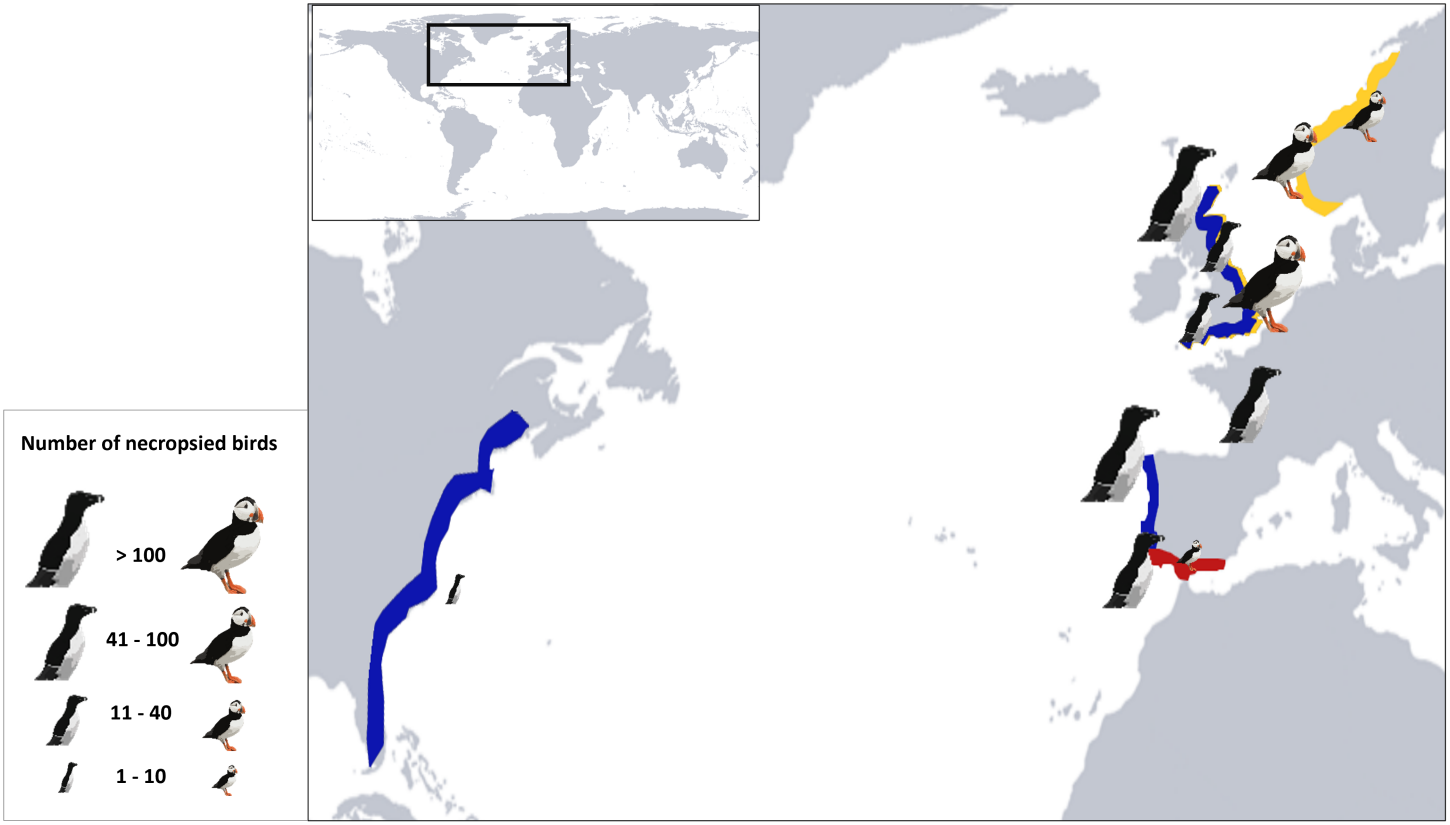
Global distribution of registered areas with strandings and necropsied Razorbill (blue line), Atlantic puffin (orange line), and our study area (red line).

The reviewed literature indicates a sparse coverage of necropsies conducted on Razorbills and Atlantic puffins globally. The majority of studies were carried out in England and Northern Europe, with additional research conducted along the East Coast of the United States and Southern Europe (Figure 8). Specifically, concerning body condition assessments, only a limited number of studies have provided data for Atlantic puffins. Anker‐Nilssen et al. (2003) visually described body condition, finding no evidence of fat deposits and observing that individuals were extremely emaciated. In contrast, Anker‐Nilssen et al. (2017) utilized a scale to score muscle, subcutaneous, and intestinal fat deposits, reporting consistently low body condition values along the Norwegian coast. These findings underscore the need for standardized protocols to assess the body condition of Razorbills and Atlantic puffins, as highlighted above.

## 4 DISCUSSION

### 4.1 Stranded alcid seabirds

Mass bird deaths often occur due to oil spills (Munilla et al., 2011). However, starvation has been seen as a primary factor for massive mortality in alcids globally (Anker‐Nilssen et al., 2003, 2017; Diamond et al., 2020; Fullick et al., 2022; Morley et al., 2016). This could also be the case in the Western Mediterranean, because during the season when stranded birds were found, environmental conditions likely could limit their food availability, as previously outlined by Jones et al. (2018) and Newell et al. (2015).

In the winter season of 2022–2023, a total of 703 stranded birds from two alcids species were documented in the western Mediterranean. This number is comparable to the 200 stranded Atlantic puffins found in Norway in 2016 (Anker‐Nilssen et al., 2017) and similar to other mass mortality events, including 62 Atlantic puffins, 626 Razorbills, and 474 Guillemots in England in February 1983 (Hope Jones et al., 1984). However, this event is much smaller than the 9148 Cassin's Auklets (*Ptychoramphus aleuticus*) reported in California in 2014–2015 (Jones et al., 2018) and significantly smaller than the 51,683 dead birds, of which 53.5% were Atlantic puffins, along the European coast in the winter season 2013–2014 (Morley et al., 2016). Most of the areas experiencing these mortalities are situated around the North Atlantic region, where a significant portion of the colonies are located (Guilford et al., 2011; Lavers et al., 2007). However, some events have also been recorded in the Mediterranean in recent years, although they were not as extensive (Belda & Sanchez, 2001; Cooper et al., 2003).

### 4.2 Metabolic state of stranded alcids and body condition

Regarding intermediary metabolism, there appears to be a common pattern in both bird species, characterized by probably low carbohydrate values (glucose and glycogen) along with high levels of lactate in muscle tissues. Although it would be important for the correct interpretation of the results to analyse the biochemical parameters evaluated from the tissues of healthy individuals used as control samples, this was not possible due to the nature of the research. However, it is known that carnivorous birds, such as the species studied here, engage in continuous gluconeogenesis primarily in the liver, using amino acids during feeding and fasting periods (Braun & Sweazea, 2008; Pollock, 2002). This process enables them to obtain glucose molecules crucial for producing high‐energy biomolecules such as ATP and NADH in the Krebs cycle through predominant aerobic glycolysis (Vock et al., 1996). Complementary, anaerobic glycolysis is fuelled by phosphorylated glucose derived from muscle glycogen, which is converted into lactate that can be accumulated in muscle during the Cory cycle (Meléndez‐Morales et al., 2009; Wynkoop et al., 2022). Our results suggested a prevalence of this anaerobic energetic pathway in most of the birds sampled in the present study, possibly indicating prolonged and strenuous exercise, where carbohydrate utilization during exercise closely matches muscle energy demands (Holloszy et al., 1998; Meléndez‐Morales et al., 2009). In contrast, fat utilization during exercise is less regulated, as there are no mechanisms for closely matching availability and metabolism to the rate of energy expenditure (Holloszy et al., 1998). Therefore, an optimum level of glucose and tissue glycogen is essential for performing prolonged and strenuous exercise, such as migration (Holloszy et al., 1998; Lupiáñez et al., 1996). The metabolite values found in the tissues of sampled birds are consistent with a state of fatigue that could result from the development of hypoglycaemia (Muneer, 2021) or depletion of muscle glycogen (Braun & Sweazea, 2008). This physiological condition could probably lead to bird mortality, as birds have naturally higher blood glucose levels compared to other vertebrates (Sweazea, 2022), due to their high metabolic rate during migratory flights, which are conducted in a fasting state (Jenni‐Eiermann, 2017).

This low body condition could be attributed to exhaustion resulting from possible disorientation and fasting conditions caused by extreme meteorological phenomena, such as the NAO (Durant et al., 2003). During the study period, fluctuations in the NAO index appeared to correlate with changes in bird migration and mortality patterns. In months with positive NAO, such as November 2022 (+0.69), January (+1.25), and February 2023 (+0.92), milder conditions were recorded in northern Europe and drier conditions in the south. These climatic conditions were associated with stronger winds and potential resource scarcity at stopover sites along the flyway. Conversely, months with negative NAO, such as December 2022 (−0.15) and March 2023 (−1.11), were characterized by colder and drier conditions in the breeding and stopover areas, possibly leading to foraging difficulties (National Centers for Environmental Information, 2024). In their study, Yao et al. (2023) identified extreme cold events associated with the NAO in November and December 2022. These findings suggest that NAO fluctuations could significantly have influenced bird migration and mortality.

For interpreting the metabolite values obtained in this assay, it is also important to consider natural post‐mortem degradation. The degradation of biological compounds such as carbohydrates and lipids is a complex process influenced by factors including temperature, humidity, microbial population, and aerobic/anaerobic conditions, as well as the specific chemical composition of the compounds (Carter et al., 2007; Forbes & Carter, 2015). This degradation process varies significantly depending on the time elapsed since the animal's death and the environmental condition (Hyde et al., 2013). Thus, although determining the exact post‐mortem chronological time of the collected individuals and its effect on the degree of organic matter decomposition was not feasible, it must be highlighted that this factor could impact the concentration of analysed tissue metabolites.

While the results presented here can provide background for future studies, it is crucial for future research to complement our analysis and determine the impact level that biotic and abiotic factors influence the presence/degradation of metabolites in the post‐mortem tissues. Considering the low body condition of the studied seabirds and the premature age of the individuals, the decline in forage fish in pre‐migration areas could also contribute to their initiation of migratory stages in weakened physical states, as suggested by Breton and Diamond in 2014.

### 4.3 Conservation implications and recommendations

Although many seabird populations are known to be endangered (Becker & Beissinger, 2006; Gaston & Descamps, 2011), after a thorough examination of the literature, it should be highlighted that no standardized protocols have been used to assess the body condition of stranded alcid seabirds.

Standardizing protocols are crucial for determining the potential causes of death in seabirds, offering a global perspective on the issue, which, in turn, might allow us to implement conservation measures to protect these species or, at the very least, minimize the impacts contributing to their decline. Therefore, we propose a body condition index that considers not only biometric measurements but also information on the physical state of the individual (the pectoral muscle, and subcutaneous and intestinal fat scores). This comprehensive approach would provide more detailed insights into the potential cause of death and facilitate global comparisons.

## AUTHOR CONTRIBUTIONS

**Yada Trapletti‐Lanti:** Data curation (equal); formal analysis (equal); methodology (equal); writing – original draft (lead). **Mónica Expósito‐Granados:** Methodology (equal); writing – review and editing (equal). **Sergio López‐Martínez:** Methodology (equal); writing – review and editing (equal). **Miguel Torres:** Formal analysis (equal); methodology (equal); writing – review and editing (equal). **Marga L. Rivas:** Conceptualization (lead); supervision (lead); writing – original draft (supporting); writing – review and editing (equal).

## CONFLICT OF INTEREST STATEMENT

The authors have no conflict of interest.

## Supporting information


Data S1.

## Data Availability

The data that support the findings of this study are openly available in the Zenodo repository at https://zenodo.org/records/10641718
