# Supplementary material for: Body condition of stranded Razorbills and Atlantic Puffins in the Western Mediterranean
Source: Ecol Evol. 2024 Sep 11;14(9):e70161. doi: 10.1002/ece3.70161 (PMC11387722; doi:10.1002/ece3.70161)
Supplement: Supplementary file 1 — Data S1. [file ECE3-14-e70161-s001.docx]

**Body condition of stranded Razorbill and Atlantic puffin in the Western Mediterranean**

**SUPPLEMENTARY MATERIAL**

Table 1 SI. Summary of measured variables for adults and juveniles Razorbills (*Alca torda*) and Atlantic Puffins (*Fratercula arctica*).

| Variables | Razorbills  n = 84 | | Razorbills Adults  n = 8 | | Razorbills Juveniles  n = 71 | | Puffins  n = 11 | | Puffins Adults  n = 3 | | Puffins Juveniles  n = 7 | |
| --- | --- | --- | --- | --- | --- | --- | --- | --- | --- | --- | --- | --- |
|  | **Mean** | **SD** | **Mean** | **SD** | **Mean** | **SD** | **Mean** | **SD** | **Mean** | **SD** | **Mean** | **SD** |
| Body mass (g) | 414.95 | 52.31 | 423.50 | 67.11 | 413.75 | 52.23 | 270.44 | 91.51 | 340.73 | 124.94 | 260.89 | 52.14 |
| Wing length (mm) | 188.95 | 4.58 | 194.00 | 2.52 | 188.33 | 4.51 | 171.24 | 30.83 | 157.67 | 7.77 | 180.09 | 36.13 |
| Tarsus length (mm) | 31.40 | 1.69 | 31.30 | 0.89 | 31.42 | 1.79 | 27.37 | 2.20 | 29.25 | 2.65 | 26.68 | 1.82 |
| Head length (mm) | 90.60 | 2.89 | 90.05 | 3.90 | 90.65 | 2.77 | 74.53 | 4.08 | 77.59 | 3.61 | 73.19 | 4.09 |
| Bill length (mm) | 30.29 | 1.55 | 30.83 | 1.71 | 30.27 | 1.48 | 37.34 | 4.00 | 40.47 | 0.58 | 35.80 | 4.47 |
| Culmen length (mm) | 20.03 | 1.29 | 19.86 | 1.45 | 20.10 | 1.25 | 20.82 | 2.15 | 21.41 | 1.97 | 20.72 | 2.52 |
| Bill width (mm) | 15.36 | 1.31 | 18.04 | 1.22 | 15.11 | 0.94 | 20.65 | 4.49 | 23.30 | 4.54 | 18.48 | 3.63 |
| Body condition Index | 1.30 | 0.55 | 1.68 | 0.70 | 1.27 | 0.52 | 1.63 | 0.84 | 2.07 | 0.73 | 1.57 | 0.93 |


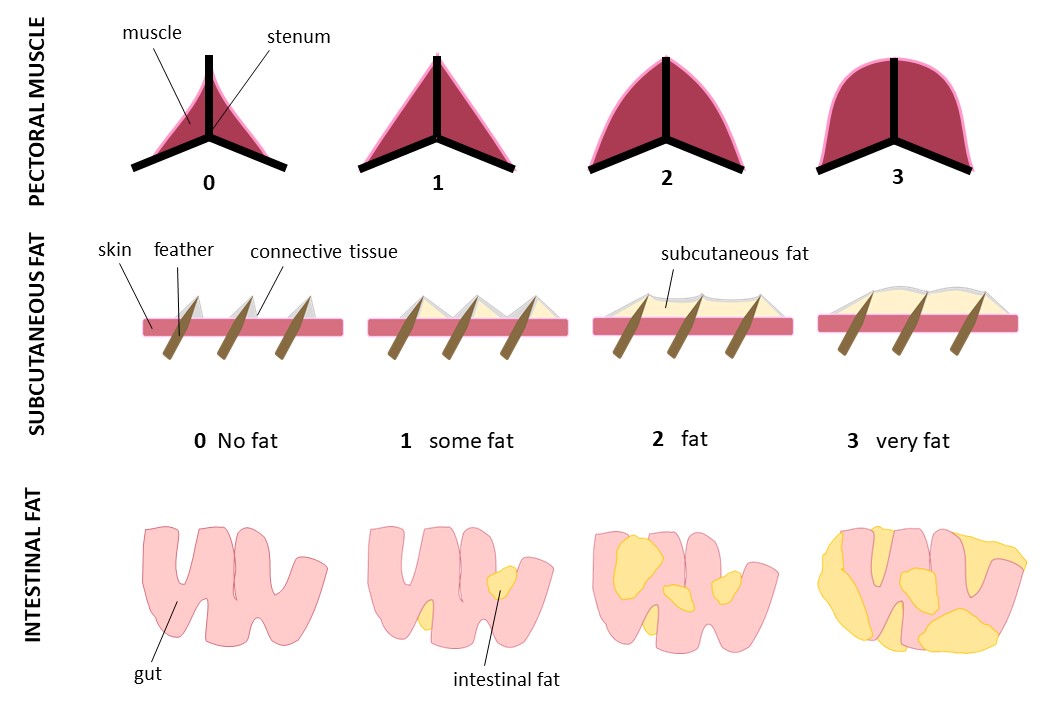


Figure 1 SI. Scoring levels to measure the development of pectoral muscles, and the presence of subcutaneous fat and intestinal fat.

Table 2 SI. Summary of the studies performing necropsies and included in the global database for Razorbills (*Alca torda*) and Atlantic Puffins (*Fratercula arctica*).

| Reference | Specie | Location | Date | Number of individuals | Body mass (g) | Age |
| --- | --- | --- | --- | --- | --- | --- |
| Morley et al., 2016 | *Alca torda* | England | March 2014 | 44 | 451.9 ± 53.9 | ND |
| Fullick et al., 2022 | *Alca torda* | Eastern Scotland and England | August-November 2021 | 29 | ND | ND |
| Simpson and Fisher, 2017 | *Alca torda* | South-west England | 1981–2016 | 13 | ND | Adults |
| Simpson and Fisher, 2017 | *Alca torda* | South-west England | 1981–2016 | 12 | ND | Juveniles |
| Diamond et al., 2020 | *Alca torda* | East coast of the US | Winter 2012/2013 | 3 | 734.5 ± 19.2 | Adults |
| Diamond et al., 2020 | *Alca torda* | East coast of the US | Winter 2012/2013 | 1 | ND | Juveniles |
| Hope Jones et al., 1984 | *Alca torda* | England | February 1983 | 401 | 441.3 ± 21.5 | Adults |
| Hope Jones et al., 1984 | *Alca torda* | England | February 1983 | 205 | 419.5 ± 23.8 | Juveniles |
| Costa et al., 2019 | *Alca torda* | Central coast of Portugal | 2012-2016 | 108 | ND | Adults |
| Costa et al., 2019 | *Alca torda* | Central coast of Portugal | 2012-2016 | 506 | ND | Juveniles |
| Our study | *Alca torda* | Western coast of Spain | November 2022 –  February 2023 | 8 | 423.5 ± 67.1 | Adults |
| Our study | *Alca torda* | Western coast of Spain | November 2022  -February 2023 | 71 | 413.7 ± 52.2 | Juveniles |
| Anker-Nilssen et al., 2018 | *Fratercula arctica* | Faroe Island | October-March 1981-2017 | 133 | 540.3 ± 8.7 | Adults |
| Anker-Nilssen et al., 2003 | *Fratercula arctica* | Central Norway | April 2002 | 28 | 336.5 ± 9.4 | Adults |
| Anker-Nilssen et al., 2017 | *Fratercula arctica* | southwest Norway | February - March 2016 | 42 | 286.0 ± 5.8 | Adults |
| Anker-Nilssen et al., 2017 | *Fratercula arctica* | southwest Norway | February - March 2016 | 31 | 261.3 ± 9.1 | Juveniles |
| Hope Jones et al., 1984 | *Fratercula arctica* | England | February 1983 | 23 | 313 ± 46.0 | Adults |
| Hope Jones et al., 1984 | *Fratercula arctica* | England | February 1983 | 27 | 291.0 ± 39.5 | Juveniles |
| Our study | *Fratercula arctica* | Western coast of Spain | November 2022 –  February 2023 | 3 | 340.7 ± 124.9 | Adults |
| Our study | *Fratercula arctica* | Western coast of Spain | November 2022 –  February 2023 | 7 | 260.9 ± 52.1 | Juveniles |

Table 3 SI. Summary of the studies selected to calculate the body condition index for Razorbills (*Alca torda*) and Puffins (*Fratercula arctica*). Species in bold belong to our study. The numbers of Muscle, Intestinal Fat and Subcutaneous Fat refers to the score of the condition of pectoral muscle and the quantity of fat (0-3).

| Specie | Number of individuals | Date | Location | Body mass (g)  Mean ± SD | Wing Length (mm)  Mean ± SD | Muscle | Intestinal Fat | Subcutaneous Fat | Age | Body Condition Index |
| --- | --- | --- | --- | --- | --- | --- | --- | --- | --- | --- |
| *Alca torda* | 8 | November 2022 - February 2023 | Western coast of Spain | 423.5 ± 67.1 | 194.0 ± 2.5 | 1 | 0 | 0 | Adults | 1.7 |
| *Alca torda* | 71 | November 2022 -February 2023 | Western coast of Spain | 413.7 ± 52.2 | 188.3 ± 4.5 | 1 | 0 | 0 | Juveniles | 1.3 |
| *Fratercula arctica* | 28 | April 2002 | Central Norway | 336.5 ± 9.4 | 172.0 ± 1.2 | 0 | 0 | 0 | Adults | 0.5 |
| *Fratercula arctica* | 42 | February - March 2016 | southwest Norway | 286.0 ± 5.8 | 161.3 ± 1.1 | 1 | 0 | 0 | Adults | 1.5 |
| *Fratercula arctica* | 31 | February - March 2016 | southwest Norway | 261.3 ± 9.1 | 152.2 ± 0.9 | 1 | 0 | 0 | Juveniles | 1.6 |
| *Fratercula arctica* | 3 | November 2022 - February 2023 | Western coast of Spain | 340.7 ± 124.9 | 157.7 ± 7.8 | 2 | 0 | 0 | Adults | 2.1 |
| *Fratercula arctica* | 7 | November 2022 - February 2023 | Western coast of Spain | 260.9 ± 52.1 | 180.1 ± 36.1 | 1 | 0 | 0 | Juveniles | 1.6 |

Table 4 SI. Kruskal-Wallis Test results in measured variables between adults and juveniles Razorbills and Atlantic Puffins.

|  | Razorbill  Adults vs. Juveniles | | | Atlantic Puffins  Adults vs. Juveniles | | |  |
| --- | --- | --- | --- | --- | --- | --- | --- |
| Variables | **Test statistic** | **df** | ***p-value*** | **Test statistic** | **df** | ***p-value*** | |
| Body Mass | 6.106e-05 | 1 | 0.993 | 0.003 | 1 | 0.957 | |
| Wing length | 12.013 | 1 | <0.001 | 1.946 | 1 | 0.163 | |
| Tarsus length | 0.451 | 1 | 0.501 | 0.544 | 1 | 0.460 | |
| Head length | 0.047 | 1 | 0.826 | 0.998 | 1 | 0.317 | |
| Bill length | 0.938 | 1 | 0.332 | 0.938 | 1 | 0.332 | |
| Culmen length | 0.574 | 1 | 0.448 | 0.0003 | 1 | 0.985 | |

Table 5 SI. Rotation matrix obtained by performing a Principal Component Analysis to select the variable to be used for body condition index.

Important loadings (>0.50) within each component are depicted in bold.

| Parameter | Loadings of Principal Components | | | | | | |
| --- | --- | --- | --- | --- | --- | --- | --- |
|  | **PC1** | **PC2** | **PC3** | **PC4** | **PC5** | **PC6** | **PC7** |
| Body mass | 0.197 | **0.504** | 0.124 | **0.805** | -0.085 | 0.188 | 0.034 |
| Wing | 0.365 | 0.418 | 0.297 | -0.261 | **0.681** | 0.269 | 0.003 |
| Tarsus | 0.159 | **0.622** | -0.455 | -0.448 | -0.359 | 0.207 | 0.091 |
| Head | 0.466 | -0.106 | -0.266 | 0.142 | -0.300 | **0.727** | -0.251 |
| Bill | 0.494 | -0.239 | -0.079 | -0.046 | 0.114 | **0.539** | **-0.622** |
| Culmen | 0.462 | -0.331 | -0.330 | 0.098 | 0.197 | 0.159 | **0.703** |
| Bill width | 0.359 | -0.084 | **0.710** | -0.225 | **-0.508** | 0.074 | 0.215 |

Table 6 SI. Eigenvalue of each Component

| Components | Eigenvalue | Percentage of variance (%) | Cumulative percentage  of variance (%) |
| --- | --- | --- | --- |
| Component 1 | 2.765 | 39.497 | 39.498 |
| Component 2 | 1.274 | 18.196 | 57.694 |
| Component 3 | 0.879 | 12.558 | 70.251 |
| Component 4 | 0.826 | 11.800 | 82.052 |
| Component 5 | 0.523 | 7.471 | 89.523 |
| Component 6 | 0.461 | 6.579 | 96.103 |

Table 7 SI. Results from random forest model.

| Parameter | Contribution of each variable to the variable “Body mass” |
| --- | --- |
| Bill width | 27133.32 |
| Culmen | 26320.68 |
| Head | 25479.06 |
| Wing | 24560.17 |
| Tarsus | 21246.37 |
| Bill | 20768.23 |
